# SLGNN: synthetic lethality prediction in human cancers based on factor-aware knowledge graph neural network

**DOI:** 10.1093/bioinformatics/btad015

**Published:** 2023-01-16

**Authors:** Yan Zhu, Yuhuan Zhou, Yang Liu, Xuan Wang, Junyi Li

**Affiliations:** School of Computer Science and Technology, Harbin Institute of Technology (Shenzhen), Shenzhen 518055, China; School of Computer Science and Technology, Harbin Institute of Technology (Shenzhen), Shenzhen 518055, China; School of Computer Science and Technology, Harbin Institute of Technology (Shenzhen), Shenzhen 518055, China; Guangdong Provincial Key Laboratory of Novel Security Intelligence Technologies, Harbin Institute of Technology (Shenzhen), Shenzhen 518055, China; School of Computer Science and Technology, Harbin Institute of Technology (Shenzhen), Shenzhen 518055, China; Guangdong Provincial Key Laboratory of Novel Security Intelligence Technologies, Harbin Institute of Technology (Shenzhen), Shenzhen 518055, China; School of Computer Science and Technology, Harbin Institute of Technology (Shenzhen), Shenzhen 518055, China; Guangdong Provincial Key Laboratory of Novel Security Intelligence Technologies, Harbin Institute of Technology (Shenzhen), Shenzhen 518055, China

## Abstract

**Motivation:**

Synthetic lethality (SL) is a form of genetic interaction that can selectively kill cancer cells without damaging normal cells. Exploiting this mechanism is gaining popularity in the field of targeted cancer therapy and anticancer drug development. Due to the limitations of identifying SL interactions from laboratory experiments, an increasing number of research groups are devising computational prediction methods to guide the discovery of potential SL pairs. Although existing methods have attempted to capture the underlying mechanisms of SL interactions, methods that have a deeper understanding of and attempt to explain SL mechanisms still need to be developed.

**Results:**

In this work, we propose a novel SL prediction method, SLGNN. This method is based on the following assumption: SL interactions are caused by different molecular events or biological processes, which we define as SL-related factors that lead to SL interactions. SLGNN, apart from identifying SL interaction pairs, also models the preferences of genes for different SL-related factors, making the results more interpretable for biologists and clinicians. SLGNN consists of three steps: first, we model the combinations of relationships in the gene-related knowledge graph as the SL-related factors. Next, we derive initial embeddings of genes through an explicit message aggregation process of the knowledge graph. Finally, we derive the final gene embeddings through an SL graph, constructed using known SL gene pairs, utilizing factor-based message aggregation. At this stage, a supervised end-to-end training model is used for SL interaction prediction. Based on experimental results, the proposed SLGNN model outperforms all current state-of-the-art SL prediction methods and provides better interpretability.

**Availability and implementation:**

SLGNN is freely available at https://github.com/zy972014452/SLGNN.

## 1 Introduction

Developing effective anticancer drugs is a high priority research topic at medical and health research institutions worldwide. Identifying biochemical pathways that can be therapeutically targeted is a key step in developing new compounds. Synthetic lethality (SL) is an interaction between genes that, when both genes are mutated, can lead to reduced cell viability or even cell death, while mutations in only one of the genes in the pair not lethal (Guo, 2016). SL can selectively treat cancer cells by identifying existing mutations and targeting their synthetic lethal partners (Zhang, 2021). Detecting suitable lethal partners conventionally relies on high-throughput experimental laboratory screening technology, commonly using RNA interference (RNAi) (Luo, 2009) followed by genome editing through CRISPR (Du *et al.*, 2017). However, such approaches have severe limitations. For example, RNAi screening is prone to off-target effects increasing the risk of clinical use (Topatana, 2020). In addition, the high cost, and relatively long-time scale limits the practical use of laboratory experiment-based screening of SL interactions.

To overcome these limitations, computational approaches are gaining considerable interest. Current computational methods can be subdivided into two categories: knowledge-based methods, and supervised machine learning (Long *et al.*, 2021). Knowledge-based approaches use prior knowledge, or assumptions, to predict SL gene pairs, mainly based on observations on known SL gene pairs. For example, DAISY (Jerby-Arnon *et al.*, 2014) used a data-driven approach to identify SL interactions within the genome, based on co-expressed but not co-mutated properties of SL gene pairs. Sinha *et al.* (2017) used genomics data from multiple sources to predict SL interactions, including mutations and copy number variations. While effective, the use of this approach is clearly limited by the need for prior information related to SL, such as insight into biological metabolic networks, gene regulation, or other know interactions between previously recognized SL gene pairs. Clearly, such strategy is suboptimal in predicting novel SL interaction.

Advances in machine-learning-based methods resulted in its successful use in tasks, such as drug repurposing and gene–disease correlation in bioinformatics. Encouraged by these results, machine-learning methods have also been applied to predict SL interactions. The method proposed by Paladugu *et al.* (2008) obtained gene features from protein–protein interaction (PPI) network and used the extracted features to train a support vector machine classifier for SL interaction prediction. Das *et al.* (2019) predicted SL interactions using multi-omics data from the Cancer Genome Atlas database using a random forest model. Subsequently, Benstead-Hume *et al.* (2019) utilized gene features derived from PPI network and the Gene Ontology database to train a random forest model for SL interaction prediction. These methods are primarily based on traditional machine-learning processes. First, the features of genes are collated from different sources of data, and then the prediction model of SL interaction is derived by machine learning. As an alternative, there are methods based on graph representation learning. These methods model SL interactions as a graph, where the gene of interest is represented as a central node and potential SL interaction partners are located at the periphery. Graph representation learning relies on an encoder–decoder paradigm, in which the encoder propagates information between nodes through the topology of the graph to obtain low-dimensional representations of node features. The decoder part uses these features to complete downstream tasks like node classification, link prediction and community discovery (Hamilton, 2020). Depending on the implementation of this concept, there are two different forms of graph representation learning: matrix factorization (MF)-based methods and graph neural network (GNN)-based methods. Huang *et al.* (2019) proposed one such method, GRSMF, that used a self-representative MF encoder to predict SL interactions. Another approach, SL^2^MF (Liu *et al.*, 2020), obtained the features of genes by factorized differential gene similarity matrices from various sources and used these to predict potential SL interactions. Liany *et al.* (2020) used collective matrix factorization (CMF) to model multiple matrices for SL interaction prediction. A drawback of MF-based methods is that they simply generate a feature for each node, without parameter sharing or using node features, effectively a form of shallow embedding (Hamilton, 2020). Alternative approaches using GNNs can effectively alleviate this limitation. Cai *et al.* (2020) modeled SL interactions into a graph and used a novel dual-drop GNN to solve the sparsity problem of interaction networks. Long *et al.* (2021) proposed a novel graph contextualized attention network, GCATSL that predicted SL interactions by aggregating gene feature graphs from different sources based on an attention mechanism.

The above GNN- and MF-based methods attempt to capture the underlying biological mechanisms of SL by modeling the similarity of gene nodes. However, this capture process needs to be improved as it has limitations in the expressive capacity of homogeneous graphs. To mitigate the impact of this problem, Wang *et al.* (2021) proposed a knowledge graph-based approach, KG4SL, and was the first to apply this in biomedicine for the prediction of SL interactions. This method utilizes the rich semantic information present in the knowledge graph to capture the underlying mechanism of SL. While KG4SL uses the underlying mechanisms of SL interaction to enhance gene embedding it does not model these from the perspective of interpretability. Specifically, it cannot attribute a perceived importance value to different entities and relationships in the knowledge graph. Thus, the embedded information remains uninterpretable. For example, biological processes, such as gene expression or gene regulation, may have different importance in distinct SL interactions. The clinical utilization of SL prediction based on machine learning is limited by the lack of information regarding its biomedical relevance, its interpretability. Consequently, novel computational approaches are needed to solve this defect (Wang, 2022).

To solve this problem, we propose an improved approach, SLGNN, for SL prediction that models the preferences of genes in distinct relationships in the knowledge graph, and thus allowing a better understanding of the underlying biological mechanisms. SLGNN is based on an assumption that SL interactions are caused by different molecular events or biological processes, such as gene regulation and co-involvement of genes in biological pathways. We refer to them as SL-related factors that lead to SL interactions. For the convenience of description, ‘factor’ in the following text refers to ‘SL-related factor’. For example, Poly (adenosine diphosphate-ribose) polymerase 1 and breast cancer 1 represent a biologically and medically relevant SL pair, with both molecules being involved in DNA repair. The recognition of the interaction between these molecules in the same biological process led to the development of the first SL-based PARP inhibitor for the treatment of cancer (Lord and Ashworth, 2017). In this example, the DNA repair process appears to be the decisive factor in SL interaction between the two genes, giving an intuitive insight into this SL mechanism. By modeling the preferences of genes for different SL-related factors, the model becomes more interpretable, giving insight into the underlying biology. The computational process underpinning SLGNN is divided into three parts: first, we model the combination of relationships in the gene-related knowledge graph as SL-related factors leading to SL interaction. Second, we obtain the initial embeddings of genes through the explicit message aggregation of the knowledge graph; third, we obtain the final gene embeddings through an SL graph constructed using known SL gene pairs and based on the factor-based message aggregation mechanism. Finally, a supervised end-to-end training model is used for SL interaction prediction. In comparisons the proposed SLGNN outperformed five other state-of-the-art methods, while providing better interpretability.

## 2 Materials and methods

In this section, we introduce the data used and the formulation of the problem. Then, we describe the details of each module of SLGNN.

### 2.1 Data description

SynLethDB (Guo *et al.*, 2016) is a database containing a large number of currently known SL gene pairs. It was derived from biochemical analysis, correlation databases and data mining. The latest version of SynLethDB contains a gene-related knowledge graph called SynLethKG. This provides a graphic representation of 36 402 SL interactions between 10 004 genes. Because the SynLethDB only contains positive samples of SL interactions, to alleviate the impact of distribution differences between positive and negative samples, we used the method utilized in KG4SL to generate negative samples with the same number as the positive numbers. SynLethKG is a SL-related biomedical knowledge graph containing 11 entities and 24 relationships related to genes. The details of SynLethKG are shown in Tables 1 and 2.

**Table 1.** Numbers of the entities in the SynLethKG

Type	No. of entities
Anatomy	400
Biological process	12 703
Cellular component	1670
Compound	2065
Disease	136
Gene	25 260
Molecular function	3203
Pathway	2069
Pharmacologic class	377
Side effect	5702
Symptom	427

**Table 2.** Numbers of the relationships in the SynLethKG

Type	No. of entities
(Gene, participates, cellular component)	97 652
(Gene, participates, biological process)	619 712
(Anatomy, expresses, gene)	617 175
(Gene, regulates, gene)	267 302
(Gene, interacts, gene)	147 638
(Disease, associates, gene)	24 328
(Gene, participates, molecular function)	110 042
(Gene, covaries, gene)	62 966
(Gene, participates, pathway)	57 441
(Disease, upregulates, gene)	7730
(Compound, causes, side effect)	139 428
(Compound, binds, gene)	16 323
(Anatomy, upregulates, gene)	26
(Disease, presents, symptom)	3401
(Disease, localizes, anatomy)	3373
(Compound, treats, disease)	752
(Disease, resembles, disease)	404
(Disease, downregulates, gene)	7616
(Compound, upregulates, gene)	19 200
(Compound, downregulates, gene)	21 526
(Compound, resembles, compound)	6266
(Pharmacologic class, includes, compound)	1205
(Compound, palliates, disease)	384
(Anatomy, downregulates, gene)	31

### 2.2 Problem formulation

**SL graph.** We intended to model SL interaction as a graph $S=\left(V,E\right),$ where V is the set of *n* genes and E is the set of SL interactions. We denote $A\in R^{n\times n}$ as the adjacency matrix of this graph. If $v_{i}$ and $v_{j}$ have a SL interaction, then, $A_{ij}$ is equal to 1, otherwise $A_{ij}$ is equal to 0, modeling interactions between genes as a SL graph.

**Knowledge graph.** KG stores the attributes of real-world entities and the correlation between these entities in the form of heterogeneous information networks. The correlations are modeled as distinct relationships (Shi *et al.*, 2017). We denote SynLethKG as $G=\left(V_{e},E_{r}\right),$ where $V_{e}$ represents the entities in KG and $E_{r}$ represents the relationships in KG. KG can be represented as a collection of triples $G=\{(h,t,r)|h,t\in V_{e},r\in E_{r}\}$, where each triple (h,t,r) represents the relationships r between head entity h and tail entity t*.* It is worth noting that we set the edges in the knowledge graph to be undirected, which means that the number of relationships in the KG is doubled.

**Task description.** Given SL graph S and SynLethKG G, our task is to automatically generate gene embeddings through SynLethKG G, then perform link prediction in the SL graph S to identify potential SL pairs.

### 2.3 Overview of SLGNN

**Fig. 1.**
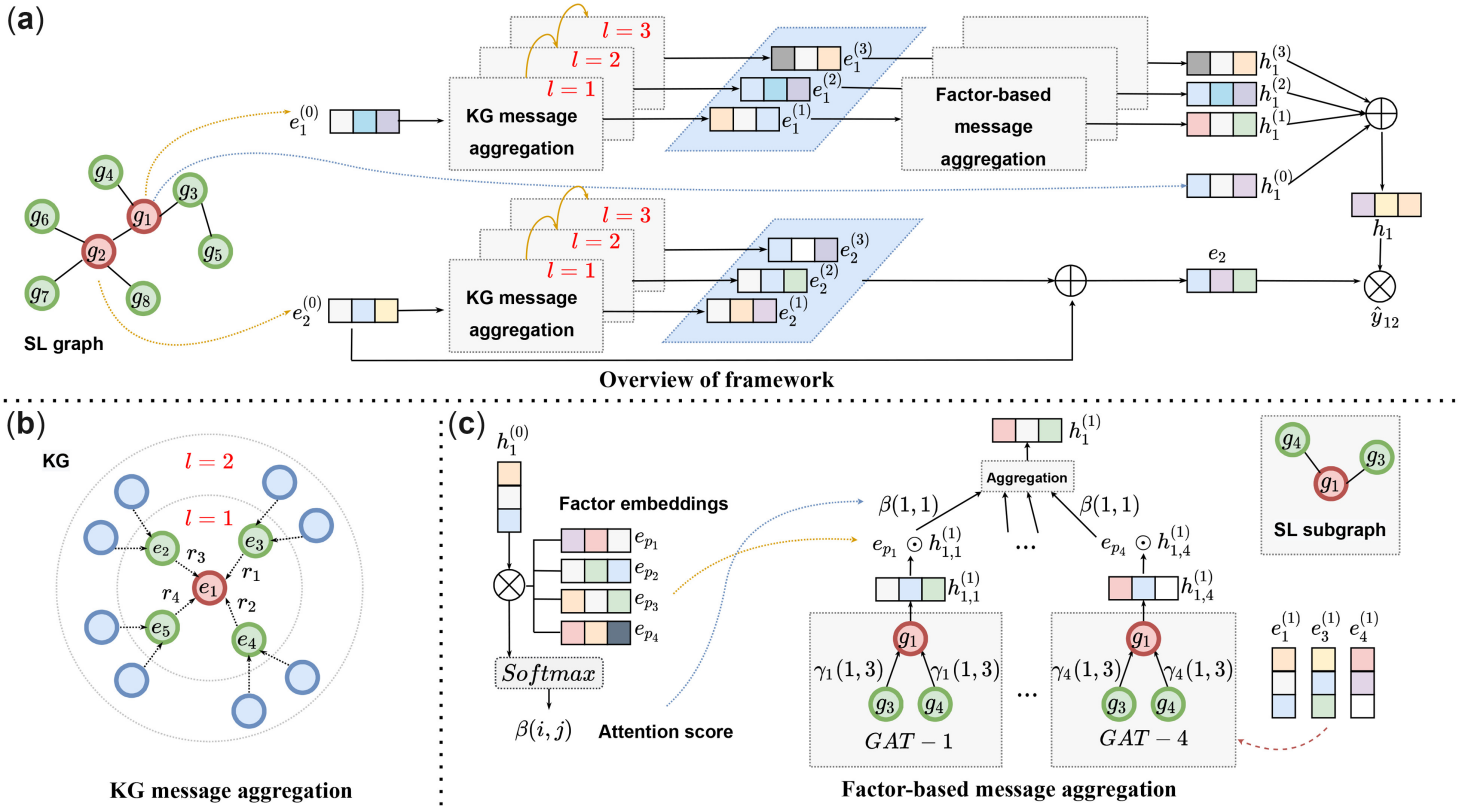
The overall architecture of SLGNN. (**a**) Overview of the framework. Here$\;g_{i\;}$represents a gene node in the SL graph and the initial gene entity embedding$\;e_{i}^{\left(0\right)}\;$is used as input. $e_{i\;}$represents the gene embedding obtained by KG message aggregation and$\;h_{i\;}$represents the gene embedding obtained by factor-based message aggregation. (**b**) KG message aggregation. The figure shows the first-order and second-order subgraphs of entity $e_{1}$ in the KG, and the neighbors are connected to $e_{1\;}$through the relationship $r_{i}$. (**c**) Factor-based message aggregation. Gene embedding $h_{1}^{\left(0\right)}$ and factor embeddings $e_{p_{i}}$ receive an attention score β through the attention mechanism. The gene embeddings obtained by KG message aggregation $e_{i}^{\left(1\right)}$are input into four different GATs to get embeddings$\;h_{1,p}^{\left(1\right)}$, where γ represents the attention score in the GAT. Finally, the gene embedding $h_{1}^{\left(1\right)}\;$is obtained by aggregation

The overall framework of the model, which consists of three main components, is illustrated in Figure 1.

**Factor modeling.** We assume that the SL interactions are caused by different SL-related factors and model these factors to enhance the interpretability of the model.

**Knowledge graph message aggregation.** Through knowledge graph convolutional networks, we perform explicit message aggregation on entity embeddings in knowledge graphs, the embeddings of genes at the knowledge graph level are obtained.

**Factor-based message aggregation.** We take the gene embedding obtained from the knowledge graph as the input and perform a message aggregation on the SL graph, incorporating the preference of a given gene for different factors into the aggregation process.

#### 2.3.1 Factor modeling

We aim to obtain SL-related factors that determine SL interaction, representing the commonness of all SL interactions. Although we can simply represent these factors as vectors, it is difficult to assign explicit biological meaning to these. Inspired by KGIN (Wang *et al.*, 2021), we model these factors as combinations of relationships in the KG. This approach derives the embeddings of factors directly from the relationships in KG, providing them with a meaning that can be explained. Suppose P is a factor set shared by all genes. Technically, for each factor p∈P, we use the following formula to derive its embedding:

(1) $$e_{p}=\sum_{r\in E_{r}}\alpha \left(r,p\right)e_{r},$$

where $e_{r}$ is the embedding of a relationship in KG, and α(r,p) is a trainable parameter. In this way, we obtain |P| different factor embeddings.

Different factors should represent independent information (Ma *et al.*, 2019). If the embedding of a factor can be represented by the embeddings of several factors, then this factor is likely to be redundant. On the contrary, if these factors are independent of each other than these they will contain more information. Here, we use distance correlation (Székely *et al.*, 2007) as a regularizer to maintain independence between factor embeddings. Distance correlation measures the correlation between two variables and equals zero if, and only if, the two variables are independent of each other. By minimizing the distance correlation between factor embeddings, the correlation between factors can be reduced, according to the following formula:

(2) $$L_{\mathrm{IND}}=\sum_{p,p^{'}\in P,p\neq p^{'}}\mathrm{dCor}\left(e_{p},e_{p^{'}}\right),$$

where dCor(⋅) is the distance correlation between two different factor embeddings:

(3) $$\mathrm{dCor}\left(e_{p},e_{p^{'}}\right)=\frac{\mathrm{dCov}\left(e_{p},e_{p^{'}}\right)}{\sqrt{\mathrm{dVar}\left(e_{p}\right)\cdot \mathrm{dVar}\left(e_{p^{'}}\right)}},$$

where dCov(⋅) is the distance covariance and dVar(⋅) is the distance variance of factor embeddings. By optimizing the above objectives, different factors can generate effective boundaries, improving the interpretability of the model.

#### 2.3.2 Knowledge graph message aggregation

We obtain the initial embedding of a gene through message aggregation of the KG, which avoids the manual design of gene features and can generate features through the rich information represented in the KG. KGNN (Lin *et al.*, 2020) used a knowledge graph convolutional network to automatically generate entity embeddings and we follow this message aggregation mechanism with slight modifications.

Relationships in KG play an important role in the knowledge graph convolution network based on the message passing mechanism, as they connect entities. A notable feature of KG is that the same entities could be linked through different relationships that correspond to distinct biological processes. Therefore, it is necessary to distinguish different relationships during message aggregation. In previous work, KGNN used an attention mechanism to model the KG relationships as decay factors, in order to control the influences of different neighbors. To enhance the performance of the model, we take a different approach to aggregate messages for different relationships in a process that is explicit:

(4) $$e_{i}^{\left(1\right)}=\frac{1}{\left|N_{i}\right|}\sum_{(r,j)\in N_{i}}e_{r}⊙e_{j}^{\left(0\right)},$$

where $N_{i}=\{(r,j)|\left(i,r,j\right)\in G\}$ is the neighbor entity set of entity i, $e_{r}$ is the embedding of relationship r, $e_{j}^{\left(0\right)}$ is the embedding of entity j and ⊙ is an element-wise product. This way, first-order neighbor messages for each entity can be aggregated. To aggregate the messages of the higher-order neighbors, we recursively set the knowledge graph convolution network to a multilayer network:

(5) $$e_{i}^{\left(l+1\right)}=\frac{1}{\left|N_{i}\right|}\sum_{(r,j)\in N_{i}}e_{r}⊙e_{j}^{\left(l\right)}.$$

Through this *l*-layer convolution network, the embeddings of entities in KG are obtained and we focus on the derived gene embedding by the sum of the representation of each layer:

(6) $$e_{i}=e_{i}^{\left(0\right)}+e_{i}^{\left(1\right)}+\cdots +e_{i}^{\left(L\right)},$$

where L is the number of layers.

#### 2.3.3 Factor-based message aggregation

After obtaining the initial gene embedding through KG message aggregation, we use a factor-based message aggregation to derive gene embeddings in the SL graph. Specifically, gene embedding $e^{\left(l+1\right)}$ of the l+1 layer of the KG convolutional network is used to obtain the corresponding gene embedding $h^{\left(l+1\right)}$.

For a given a gene, individual factors make a different contribution to the SL interaction associated with it. We use an attention mechanism to measure the importance of these factors for a given gene. For gene i∈V, the attention score β(i,p) is:

(7) $$\beta \left(i,p\right)=\frac{\exp\left(e_{p}^{T}h_{i}^{\left(l\right)}\right)}{\sum_{p^{'}\in P}\exp\left(e_{p^{'}}^{T}h_{i}^{\left(l\right)}\right)},$$

where $e_{p}$ is the embedding of factor p and $h_{i}^{\left(l\right)}$ is the embedding of the l-th layer obtained by gene i after the factor-based message aggregation, while $h_{i}^{\left(0\right)}$ is a randomly initialized embedding.

Intuitively, gene i should generate different embeddings for each factor before message aggregation. Therefore, we use graph attention networks (GATs) (Veličković *et al.*, 2017) to aggregate messages in the SL graph, adaptively deriving different gene embeddings. We input the gene embeddings of layer l+1 of the knowledge graph convolutional network into |P| different GATs, deriving the $h_{i,p}^{\left(l+1\right)}$ corresponding to the layer l+1:

(8) $$h_{i,p}^{\left(l+1\right)}=\sum_{j\in N_{i}}\gamma_{p}\left(i,j\right)e_{j}^{\left(l+1\right)},$$

(9) $$\gamma_{p}\left(i,j\right)=\frac{\exp(\sigma (a_{p}^{T}[{W_{p}e}_{i}^{\left(l+1\right)}||{W_{p}e}_{j}^{\left(l+1\right)}]))}{\sum_{k\in N_{i}}\exp(\sigma (a_{p}^{T}[{W_{p}e}_{i}^{\left(l+1\right)}||{W_{p}e}_{k}^{\left(l+1\right)}])},$$

where $\gamma_{p}\left(i,j\right)$ is the attention score in GAT-p, $N_{i}=\left\{j|\left(i,j\right)\in S\right\}\;$is the neighbor set of gene i in SL graph, $e_{j}^{\left(l+1\right)}$ is the embedding of gene j obtained by l+1 layer of knowledge graph convolution network, $W_{p}$ is a projection matrix, $a_{p}$ is a weight vector that is different for each factor and σ is an activation function, here, we use LeakyReLU. Finally, we further aggregate these gene embeddings generated by the GATs based on the attention scores of factors:

(10) $$h_{i}^{\left(l+1\right)}=\sum_{p\in P}\beta \left(i,p\right)e_{p}⊙h_{i,p}^{\left(l+1\right)},$$

where $h_{i,p}^{\left(l+1\right)}$ is the embedding of gene i obtained by GAT-p, $e_{p}\;$is the embedding of factor p, ⊙ is the element-wise product and $\beta \left(i,p\right)\;$is the attention score of gene i to factor p. Following the same strategy used for factor modeling, we attempt to make different GAT-generated embeddings independent of each other to express more information:

(11) $$L_{\mathrm{GAT}}=\sum_{p,p^{'}\in P,p\neq p^{'}}\mathrm{dCor}(h_{p}^{\mathrm{avg}},h_{p^{'}}^{\mathrm{avg}}),$$

where $h_{p}^{\mathrm{avg}}$ represents the average embedding of all genes obtained by GAT-p. Finally, we add each layer of gene embedding to obtain the final gene embedding $h_{i}$:

(12) $$h_{i}=h_{i}^{\left(0\right)}+h_{i}^{\left(1\right)}+\cdots +h_{i}^{\left(L\right)},$$

where L is the number of layers.

### 2.4 Model optimization

The inner product of the two embeddings $h_{i}$ and $e_{j}$ for the gene pair i, j is used as the probability of a SL interaction. The cross-entropy is calculated using the probability value${\;\hat{y}}_{ij}$ and the truth label:

(13) $${\hat{y}}_{ij}=\sigma ((h_{i}{)}^{T}e_{j}),$$

(14) $$L_{\mathrm{BCE}}=-\frac{1}{\left|E_{t}\right|}\sum_{i,j\in E_{t}}y_{ij}\cdot \ln({\hat{y}}_{ij})+(1-y_{ij})\cdot \ln(1-{\hat{y}}_{ij}),$$

where σ is sigmoid function. $E_{t}$ is the set of SL interactions in the training set.

Independence losses $L_{\mathrm{IND}},\;L_{G\mathrm{AT}}\;$and *L*2-normalization losses of the model parameter $L_{\mathrm{norm}}\;$must also be considered. Regularization parameters include the embeddings of entities, the embeddings of relationships and the projection matrixes. The final loss function is defined as:

(15) $$L=L_{\mathrm{BCE}}+\lambda_{1}(L_{\mathrm{IND}}+L_{\mathrm{GAT}})+\lambda_{2}L_{\mathrm{norm}},$$

where $\lambda_{1}$ and $\lambda_{2}$ are two hyperparameters that control the independence loss and the *L*2-normalization loss, respectively.

## 3 Results

In this section, we first introduce the state-of-the-art baseline methods and the implementation details, followed by parameter sensitivity analysis and model ablation study. Finally, we give an example of model interpretability.

### 3.1 Experimental setups

#### 3.1.1 Baseline methods

To validate the performance of SLGNN, we compare it to some recently published state-of-the-art SL interaction prediction methods. The first and second of these are based on MF, the third and fourth on graph convolution network, while the final one represents a knowledge graph convolution network. Note that, the first four methods do not use KGs to generate gene embeddings:

- SL^2^MF (Liu *et al.*, 2020) predicts SL interactions based on logical MF.
- CMF (Liany *et al.*, 2020) uses CMF to model multiple matrices.
- DDGCN (Cai *et al.*, 2020) predicts sparse SL interaction based on dual-dropout GCN.
- GCATSL (Long *et al.*, 2021) adopts a context-based attention network for SL prediction.
- KG4SL (Wang *et al.*, 2021) represents the first KG enhanced SL interaction prediction model.

#### 3.1.2 Implementation details

The dataset was randomly divided into training set, validation set and test set at a ratio of 8:1:1. To make the experimental result more convincing, we used 5-fold cross-validation for all methods. For all baselines, we used the hyperparameters and model parameters described in the original paper. Our proposed SLGNN was implemented in Python3.9 and PyTorch1.10. The GATs were implemented using the DGL (Wang *et al.*, 2019) framework, and parameters, such as dropout rate and negative slope, were set according to default settings in the original GAT paper. The model learning rate was set to 0.002 and an early stopping strategy was used. Due to memory constraints, we randomly dropout edges in the KG with a ratio of 0.5. In the KG, we used word embeddings in PyTorch to obtain unique embeddings for entities and relationships, and a random initialization applying a standard normal distribution.

A greedy strategy was used to confirm the optimal hyperparameters. At first, initial hyperparameters were set from related experience, then, each hyperparameter was optimized one by one. Specifically, the coefficients of constraints $\lambda_{1}\;$and $\lambda_{2}$ were tuned in {10^−5^, 10^−4^, 10^−3^, 10^−2^}, the number of GNN layers was tuned in {1, 2, 3}, the number of factors was tuned in {1, 2, 4, 8} and the dimension size was tuned in {32, 64, 128, 256}.

### 3.2 Performance evaluation

We compare the performance of SLGNN with the five previously described baseline models according to three evaluation metrics: AUC, AUPR and *F*1-score. As shown in Table 3, SLGNN outperforms all baseline methods. The AUC, AUPR and *F*1-score of SLGNN is 0.9635, 0.9710 and 0.9089, respectively, i.e. 2.1%, 1.8% and 2.9% higher than the second-best model. Compared to MF methods with shallow embedding, GNN-based methods take advantage of the topological structure of the graph to propagate information, and exploit the similarity of known SL interactions, thus improving model performance. The comparisons indicate that the two KG-based methods, KG4SL and our proposed SLGNN, exhibit greatly improved performance as they benefit from the enhanced embedding facilitated by the rich semantic knowledge present in the KG. However, to generate gene embeddings, KG4SL only uses an attention mechanism to distinguish different relationships in gene subgraphs, without considering the semantic information of relationship embeddings. From the comparisons carried out it is clear that SLGNN circumvents this limitation and performs better at generating gene embeddings for the purposes of SL interaction prediction.

**Table 3.** Comparing the performance of the different methods (the best result is shown in bold and the second-best result is underlined)

Methods	AUC	AUPR	*F*1
SL^2^MF	0.7912 ± 0.0024	0.8637 ± 0.0054	0.7478 ± 0.0077
CMF	0.8023 ± 0.0035	0.8422 ± 0.0042	0.7565 ± 0.0016
DDGCN	0.8413 ± 0.0089	0.8851 ± 0.0065	0.8133 ± 0.0065
GCATSL	0.9214 ± 0.0022	0.9450 ± 0.0033	0.8631 ± 0.0076
KG4SL	0.9436 ± 0.0019	0.9539 ± 0.0008	0.8833 ± 0.0017
SLGNN	**0.9635 ± 0.0017**	**0.9710 ± 0.0010**	**0.9089 ± 0.0017**

### 3.3 Parameter sensitivity

**Fig. 2.**
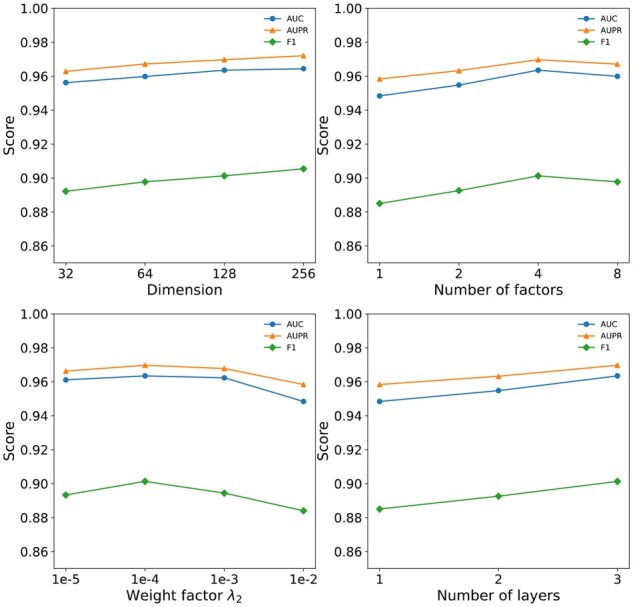
The effect of various hyperparameters of the model. This figure shows the impact of four hyperparameters on model performance, including the embedding dimension, the number of factors, the number of layers and the weight factor $\lambda_{2}$ controlling *L*2-normalization

In this section, we explore the influence of some parameters on the performance of the model. Though experiments, SLGNN achieves the best results when the number of GNN layers is 3, the number of factors is 4, the weight factor $\lambda_{1}$ is 10^−4^, $\lambda_{2}$ is 10^−3^ and the embedding dimension is 256. Therefore, we use these optimal hyperparameters as the default setting while performing sensitivity analyses on each hyperparameter. As illustrated in Figure 2, the performance of the model increases with an increasing dimension setting. A higher number of factors initially improve performance that reach its peak at four. Increasing the number further results in a slow decline in the performance. Intuitively, as the number of network layers increases, the gene entities in the knowledge graph can perceive entity information that is farther away, helping to improve model performance. The result shown in Figure 2 is consistent with this intuition. Regarding the *L*2-normalization parameter $\lambda_{2}$, it can be seen from the figure that the model performance initially improved with increasing weight factor, reach its optimum, and then start to decline.

We also explore the influence of the hyperparameter $\lambda_{1}$ controlling independence loss. As shown in Table 4, when $\lambda_{1}$ increases, the distance correlation between factor embeddings also grows. This is also accompanied by a slight decrease of the AUC, indicating that when the factors are closer to each other they contained more semantic information, improving model performance.

**Table 4.** The impact of independence between the factors

$\lambda_{1}$	AUC	AUPR	Distance correlation
10^−5^	0.9598 ± 0.0024	0.9656 ± 0.0016	2.3653
10^−4^	0.9612 ± 0.0035	0.9688 ± 0.0019	1.0275
10^−3^	0.9635 ± 0.0017	0.9710 ± 0.0010	0.0245
10^−2^	0.9622 ± 0.0013	0.9697 ± 0.0012	0.0043

### 3.4 Ablation study

In this section, we explore the relative contribution of individual components to the performance of the model, by eliminating individual components one by one.

First, we verify the influence of factor modeling. To do so, we create a variant of the model, SLGNN_w/o F_ in which the factor modeling component is removed resulting in the direct use of GAT to aggregate messages in the SL graph. When the SLGNN_w/o F_ is compared to the original model, it results a significant reduction in model performance. As shown in Table 5, the altered model is inferior to KG4SL, clearly indicating the contribution of factor modeling.

**Table 5.** Comparison of SLGNN and model variants

Model	AUC	AUPR	*F*1
SLGNN	0.9635 ± 0.0017	0.9710 ± 0.0010	0.9089 ± 0.0017
SLGNN_w/o F_	0.9345 ± 0.0012	0.9422 ± 0.0041	0.8715 ± 0.0032
SLGNN_w/o G_	0.9558 ± 0.0021	0.9664 ± 0.0013	0.8915 ± 0.0016

Next, we explore the importance of GATs to the performance of the model. Similarly, we create another variant of the model SLGNN_w/o G_, which does not use GATs in the factor-based message aggregation process. As shown in Table 5, the performance of this variant to the model is also inferior, clearly indicating that our strategy of using GAT to generate different gene embeddings with preserved independence is an effective way to get a better performance.

### 3.5 Interpretability of SLGNN

In this section, we explain the semantics of factors used in the model and give an example to illustrate the interpretability of result obtained by SLGNN.

**Fig. 3.**
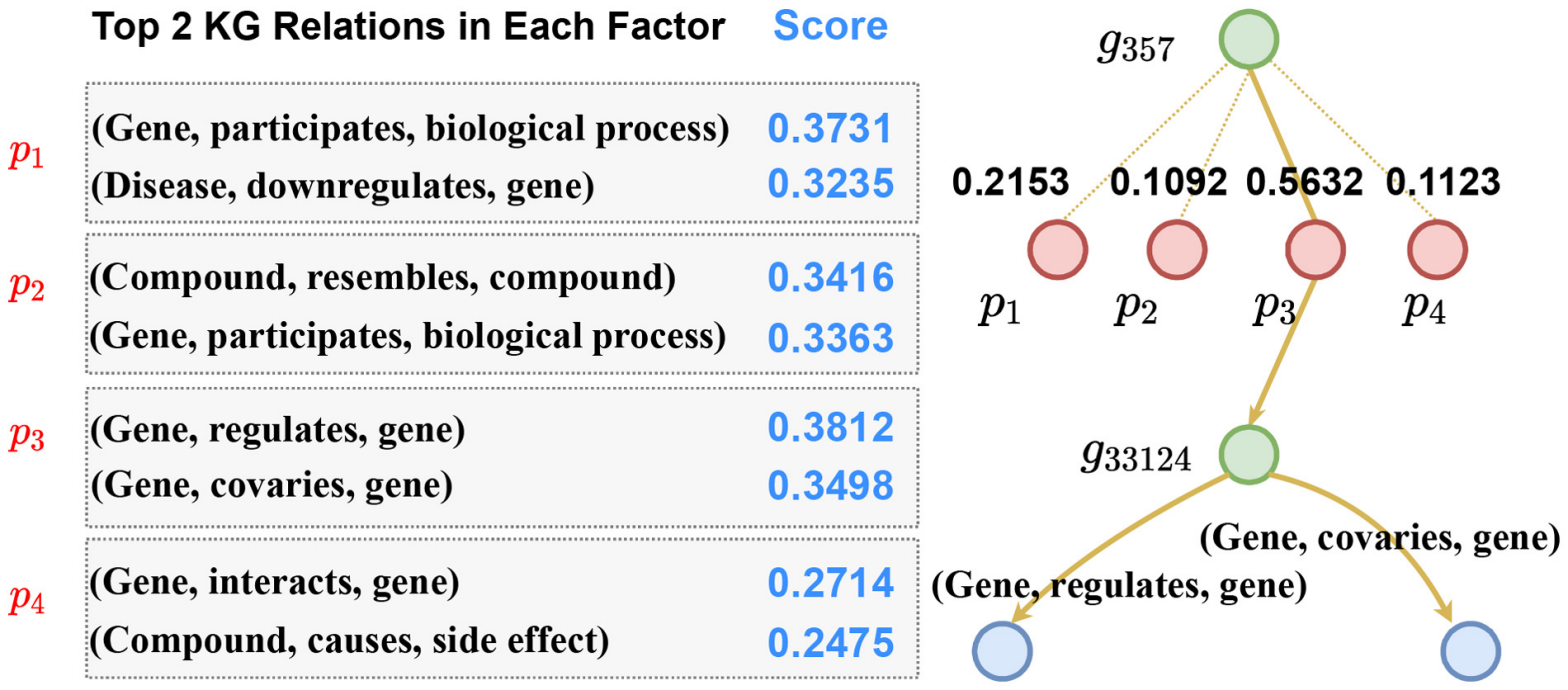
An example of SGNN interpretability. The top-2 KG relationships and their weights in the factors are shown on the left, an example of SL interactions between gene pairs indicated by factors is shown on the right

It is readily apparent that not all factors influence an interaction that results in SL. Thus, various combinations of factors in the model are weighted differently to reflect the importance of distinct factors. Of the possible relationships illustrated in Figure 3, in the composition of factor *p_1_* two particular relationships (gene, participates, biological process) and (disease, downregulates, gene) carry the highest weights. This suggests that relationships describing the interaction between genes and disease and the participation of genes in biological processes are the most likely to contain a *factor* leading to SL. As can be seen from the figure, by maintaining the independence between the factors, the KG relationship with the highest weight is different in each factor, improving the interpretability of the model.

We can explain the interpretability of our proposed model from another perspective. The importance of factors leading to SL interactions differs for each gene. In other words, genes have certain bias to given factors. As can be seen from the figure, gene_357_ and gene_33 124_ result in a SL interaction that can be derived through the attention mechanism. In this example, factor *p_3_* has the highest attention score indicating that *p_3_* is the main factor that causes the SL interaction between gene_357_ and gene_33 124_. From the semantics of factor *p_3_*, we can explore the specific mechanism of SL interaction between these two genes.

## 4 Conclusion and discussion

In this article, we propose a knowledge graph-based model SLGNN for the prediction of SL interactions, while also modeling the SL-related factors that lead to SL interactions to improve the biological interpretability of the results. First, the factors leading to SL interaction are modeled as weighted sums of different relationships in KG, and the independence of factors is maintained by minimizing the distance correlation between them. Then, the GNN-based message aggregation mechanism is used to obtain initial embeddings for the genes in KG. Finally, we conduct a factor-based message aggregation through the SL graph and use GATs to improve the performance of the model. The experimental results show that our proposed SLGNN is superior to five existing state-of-the-art methods in predicting SL interactions.

Despite its remarkable performance, SLGNN still has some limitations. For example, gene embeddings obtained through the KG graph is coupled and this has an impact on both interpretability and performance. To further improve this drawback in future work, we will attempt to use the decoupling representation learning, similar to that described in DisenKGAT (Wu *et al.*, 2021), to achieve higher quality gene embeddings. In addition, due to the sparsity of the SL graph, negative samples were created using theoretical considerations and may contain potential positive samples. In this work, we simply generated negative samples by randomly sampling. This strategy is essentially unreliable and its use in the training of the model may reduce the accuracy of predictions. Adopting alternative strategies, such as contrastive learning, may alleviate the problem of the imbalance between positive and negative samples, improving quality of negative sampling (Jiang *et al.*, 2021). Obtaining reliable negative samples is one of our future research goals.

## Funding

This work was supported by the grants from the National Key R&D Program of China [2021YFA0910700]; Shenzhen Science and Technology University Stable Support Program [GXWD20201230155427003-20200821222112001]; Shenzhen Science and Technology Program [JCYJ20200109113201726]; Guangdong Basic and Applied Basic Research Foundation [2021A1515012461, 2021A1515220115]; and Guangdong Provincial Key Laboratory of Novel Security Intelligence Technologies [2022B1212010005].

*Conflict of Interest*: none declared.

## References

[btad015-B1] Benstead-Hume G. et al (2019) Predicting synthetic lethal interactions using conserved patterns in protein interaction networks. PLoS Comput. Biol., 15, e1006888. 30995217 10.1371/journal.pcbi.1006888 PMC6488098

[btad015-B2] Cai R. et al (2020) Dual-dropout graph convolutional network for predicting synthetic lethality in human cancers. Bioinformatics, 36, 4458–4465. 32221609 10.1093/bioinformatics/btaa211

[btad015-B4] Das S. et al (2019) DiscoverSL: an R package for multi-omic data driven prediction of synthetic lethality in cancers. Bioinformatics, 35, 701–702. 30059974 10.1093/bioinformatics/bty673 PMC6378931

[btad015-B5] Du D. et al (2017) Genetic interaction mapping in mammalian cells using CRISPR interference. Nat. Methods, 14, 577–580. 28481362 10.1038/nmeth.4286 PMC5584685

[btad015-B6] Guo J. et al (2016) SynLethDB: synthetic lethality database toward discovery of selective and sensitive anticancer drug targets. Nucleic Acids Res., 44, D1011–D1017. 26516187 10.1093/nar/gkv1108 PMC4702809

[btad015-B7] Hamilton W.L. (2020) *Graph Representation Learning*. In: *Synthesis Lectures on Artificial Intelligence and Machine Learning*. Vol. **14**, Springer, Cham. pp. 1–159.

[btad015-B8] Huang J. et al (2019) Predicting synthetic lethal interactions in human cancers using graph regularized self-representative matrix factorization. BMC Bioinformatics, 20, 1–8. 31870274 10.1186/s12859-019-3197-3 PMC6929405

[btad015-B9] Jerby-Arnon L. et al (2014) Predicting cancer-specific vulnerability via data-driven detection of synthetic lethality. Cell, 158, 1199–1209. 25171417 10.1016/j.cell.2014.07.027

[btad015-B10] Jiang Z. et al (2021) Improving contrastive learning on imbalanced data via open-world sampling. In: *Advances in Neural Information Processing Systems,* Virtual Conference, December 6-14, 2021. Vol. 34, pp. 5997–6009.

[btad015-B11] Liany H. et al (2020) Predicting synthetic lethal interactions using heterogeneous data sources. Bioinformatics, 36, 2209–2216. 31782759 10.1093/bioinformatics/btz893

[btad015-B12] Lin X. et al (2020) KGNN: knowledge graph neural network for drug-drug interaction prediction. In: *Proceedings of the Twenty-Ninth International Jiont Conference on Artificial Intelligence, IJCAI-20, Yokohama, Japan*. pp. 2739–2745.

[btad015-B13] Liu Y. et al (2020) SL^2^MF: predicting synthetic lethality in human cancers via logistic matrix factorization. IEEE/ACM Trans. Comput. Biol. Bioinform., 17, 748–757. 30969932 10.1109/TCBB.2019.2909908

[btad015-B14] Long Y. et al (2021) Graph contextualized attention network for predicting synthetic lethality in human cancers. Bioinformatics, 37, 2432–2440. 10.1093/bioinformatics/btab110 33609108

[btad015-B15] Lord C.J. , Ashworth A. (2017) PARP inhibitors: synthetic lethality in the clinic. Science, 355, 1152–1158. 28302823 10.1126/science.aam7344 PMC6175050

[btad015-B9307567] Luo,J. et al (2009) A genome-wide rnai screen identifies multiple synthetic lethal interactions with the ras oncogene. Cell, 137, 835–848. 10.1016/j.cell.2009.05.006. 19490893 PMC2768667

[btad015-B16] Ma J. et al (2019) Disentangled graph convolutional networks. In: *Proceedings of the 36th International Conference on Machine Learning, Long Beach, California, USA*. pp. 4212-4221. Long Beach, California, USA.

[btad015-B17] Paladugu S.R. et al (2008) Mining protein networks for synthetic genetic interactions. BMC Bioinformatics, 9, 1–14. 18844977 10.1186/1471-2105-9-426 PMC2577120

[btad015-B18] Shi C. et al (2017) A survey of heterogeneous information network analysis. IEEE Trans. Knowl. Data Eng., 29, 17–37. 10.1109/TKDE.2017.2733530 PMC5726307 29242698

[btad015-B19] Sinha S. et al (2017) Systematic discovery of mutation-specific synthetic lethals by mining pan-cancer human primary tumor data. Nat. Commun., 8, 1–13. 28561042 10.1038/ncomms15580 PMC5460027

[btad015-B20] Székely G.J. et al (2007) Measuring and testing dependence by correlation of distances. Ann. Stat., 35, 2769–2794.

[btad015-B21] Topatana W. et al (2020) Advances in synthetic lethality for cancer therapy: cellular mechanism and clinical translation. J. Hematol. Oncol., 13, 1–22. 32883316 10.1186/s13045-020-00956-5 PMC7470446

[btad015-B22] Veličković P. et al (2018) Graph attention networks. In: *Proceedings of the International Conference on Learning Representations, Vancouver, BC, Canada*.

[btad015-B23] Wang J. et al (2022) Computational methods, databases and tools for synthetic lethality prediction. Brief. Bioinform., 23, bbac106. 35352098 10.1093/bib/bbac106 PMC9116379

[btad015-B24] Wang M. et al (2019) Deep graph library: a graph-centric, highly-performant package for graph neural networks. arXiv preprint arXiv:1909.01315.

[btad015-B25] Wang S. et al (2021) KG4SL: knowledge graph neural network for synthetic lethality prediction in human cancers. Bioinformatics, 37, i418–i425. 34252965 10.1093/bioinformatics/btab271 PMC8336442

[btad015-B26] Wang X. et al (2021) Learning intents behind interactions with knowledge graph for recommendation. In: *Proceedings of the Web Conference 2021, New York, NY, USA*, pp. 878–887.

[btad015-B27] Wu J. et al (2021) DisenKGAT: knowledge graph embedding with disentangled graph attention network. In: *Proceedings of the 30th ACM International Conference on Information & Knowledge Management, Gold Coast, Queensland, Australia*, pp. 2140–2149.

[btad015-B28] Zhang B. et al (2021) The tumor therapy landscape of synthetic lethality. Nat. Commun., 12, 1–11. 33627666 10.1038/s41467-021-21544-2 PMC7904840

